# Prevalence of Intestinal Parasites and *Schistosoma mansoni* and Associated Factors among Fishermen at Lake Tana, Northwest Ethiopia

**DOI:** 10.1155/2021/4534689

**Published:** 2021-11-30

**Authors:** Abebaw Fentahun, Tadesse Hailu, Getaneh Alemu

**Affiliations:** ^1^Mertule Mariam Primary Hospital, East Gojjam Zone, Amhara Region, Ethiopia; ^2^Department of Medical Laboratory Science, Bahir Dar University, Bahir Dar, Ethiopia

## Abstract

**Background:**

Intestinal parasites and *Schistosoma mansoni* infections adversely affect the health of humans in the world especially in sub-Saharan African countries including Ethiopia. Fishermen who spend most of their time in water bodies are supposed to be at high risk of schistosomiasis and other water-born parasites. However, the magnitude of these parasitic infections and their determinant factors are not well addressed.

**Methods:**

A cross-sectional study was conducted at Lake Tana among 388 fishermen from March to May 2021. Questionnaire data was collected through face to face interview. Stool sample from each participant was processed by the Kato-Katz and spontaneous tube sedimentation techniques. Data were analyzed using SPSS version 26.

**Results:**

One hundred sixty four (42.3%) and 88 (22.7%) participants were infected by intestinal parasites and *Schistosoma mansoni*, respectively. One hundred twenty two (31.4%) and 42 (10.8%) participants were infected by soil-transmitted helminths and intestinal protozoa, respectively. Attending only primary school (AOR = 2.02, *p* = 0.014) or being illiterate (AOR = 2.54, *p* = 0.004) and not washing hands before meal (AOR = 2.23, *p* = 0.007) were significantly associated with intestinal parasitic infections. Illiterate educational status (AOR = 2.37, *p* = 0.022), fishing by bargee (AOR = 2.43, *p* = 0.005), fishing ≥4 days per week (AOR = 2.27, *p* = 0.029), swimming habit (AOR = 3.03, *p* = 0.030), and participation in irrigation (AOR = 3.09, *p* ≤ 0.001) predispose fishermen to *S. mansoni* infection.

**Conclusion:**

Intestinal parasites and *S. mansoni* infections are highly prevalent among fishermen at Lake Tana basin. Low education level and frequent water contact predispose fishermen for intestinal parasites and *S. mansoni*. Therefore, health education to fishermen on intestinal parasites and *S. mansoni* infection and regular deworming should be advocated.

## 1. Introduction

Intestinal parasitic infections (IPIs) are major public health problems in developing countries [1]. An estimated 3.5 billion people are infected, and 450 million people are ill due to intestinal parasites (IPs) worldwide [2]. Among the intestinal helminth, soil-transmitted helminths (STH) comprising *Ascariasis lumbricoides*, hookworms, and *Trichiuris trichiura* accounted the highest prevalence in sub-Saharan Africa [3, 4]. Lack of proper sanitation and hygiene, the habit of eating raw vegetables and not washing hands before meal, lack of provision of safe water, open defecation, and insufficient health care are the risk factors for the high prevalence of IPIs [1, 2, 5, 6].

Not only IPIs but also schistosomiasis, caused by the genus *Schistosoma*, is the most neglected parasitic diseases in sub-Saharan countries. Schistosomiasis affects over 250 million people, and 780 million people are at risk of infection worldwide, of which more than 90% of the infections occur in Africa notably in sub-Saharan Africa [7–9]. The global disease burden of schistosomiasis is estimated to be 3.5 million disability-adjusted life years lost [8, 10]. *S. mansoni* and *S. heamatobium* are the two species found in Ethiopia with *S. mansoni* being the most prevalent species in the country [11, 12]. In Ethiopia, about 5.01 million peoples are infected and 37.5 million are at risk of the disease schistosomiasis [11, 13]. Fishing and irrigation activities, existence of snails, washing clothes and fetching water in infested water, open defecation practice, and being fishermen are the predisposing factors for schistosomiasis [7, 8, 13–16].

Fishermen who are performing their day to day activities in the water bodies are believed to be at high risk of *S. mansoni* infection. In the present study area, there are many people who are living by fishing in Lake Tana which is a potential infectious area for schistosomiasis. In addition, open defecation is a common problem around the Lake where fishermen are performing their daily activities due to the absence of community latrine. Possibility of acquiring IPs and *S. mansoni* infections is expected to be high due to the above risk factors; however, there is a scarcity of information about IPs and *S. mansoni* infection rate and their associated factors among fishermen who are working in Lake Tana. Therefore, the aim of this study was to assess the prevalence and associated factors of IPs and *S. mansoni* infections among fishermen in Bahir Dar zuria and Dera district kebeles bordering the Lake Tana.

## 2. Materials and Methods

### 2.1. Study Design, Area, and Period

A cross-sectional study was conducted in Bahir Dar zuria and Dera district kebeles around the Lake Tana basin from March to May 2021. Bahir Dar zuria district is located around Bahir Dar town, the capital city of Amhara Regional State, 565 km northwest of Addis Ababa. The mean altitude of the district is 1,800 m above sea level and the temperature of the district ranges from 10 to 32°C with annual rainfall of 800-1250 mm [17, 18]. Dera district is located in South Gondar administrative zone, 602 km away from Addis Ababa. The mean altitude of the district is 2077 m above sea level. The area's mean annual rain fall and mean annual temperature were 1300 mm and 26°C, respectively.

### 2.2. Sample Size Calculation and Selection of Study Participants

The sample size (*n*) was calculated using single population proportion formula. Since there is no previous data conducted in the study area, we used a prevalence of 50% (*p* = 0.5), 95% confidence level (*Z* *α*/2 = 1.96), and 5% margin of error (*d* = 0.05) to calculate the sample size.

(1) $$\begin{array}{c} n={\left(\frac{Z\alpha }{2}\right)}^{2}\;\frac{\left(P\right)\left(1-P\right)}{d^{2}}={\left(1.96\right)}^{2}\frac{\left(0.5\right)\left(1-0.5\;\right)}{{\left(0.05\right)}^{2}}=384. \end{array}$$

After adding 10% (38) for nonrespondents, the final sample size was 422. Among the nine districts bordering Lake Tana two districts (Bahir Dar zuria and Dera district) were selected by simple random sampling technique. The study participants in each district were proportionally allocated by considering the total number of fishermen in each district. So, 308 fishermen from a total of 598 fishermen from Bahir Dar zuria district and 114 fishermen from a total of 220 fishermen from Dera district were included. Study participants were selected by systematic random sampling technique from the sample frame of the lists of fishermen in the fishermen association registration book. Fishermen in Bahir Dar zuria and Dera districts who were actively engaged in fishing activities for the last six weeks during the study period and volunteered to participate were included in the study. Fishermen who had taken treatment for intestinal parasites and *S. mansoni* infection two months prior to or during the data collection time were excluded from the study.

### 2.3. Data Collection and Processing

A structured questionnaire was used to obtain data on sociodemographic characteristics and factors associated with IPIs and intestinal schistosomiasis by trained laboratory technicians. Approximately, five grams of fresh stool sample was collected using labeled, clean, dry, and leak-proof container in a nearby toilet. The stool specimen was transported to Bahir Dar University, College of Medicine and Health Sciences, parasitology laboratory following specimen transport guideline for parasitological examination. The stool specimen was processed using the Kato-Katz (KK) and spontaneous tube sedimentation techniques (STST).

In KK technique, a stool sample was pressed through a sieve to remove large particles. About 41.7 milligrams of sieved stool was transferred to the template. Then, the template was removed, and the stool sample was covered with cellophane (previously immersed with glycerol-malachite green for 24 hrs) and pressed with a new slide. Two KK thick smears were prepared from each participant's sample and examined for intestinal helminths and *S. mansoni* detection. Infection intensity was determined for STHs and *S. mansoni* based on the number of eggs per gram of stool (EPG) [15, 19, 20]. The intensity was categorized as light (1–4999 EPG), moderate (5000–49999 EPG), and heavy (≥50,000 EPG) for *A. lumbricoides*; light (1–1999 EPG), moderate (2000–3999 EPG), and heavy (≥4000 EPG) for Hookworms; light (1–999 EPG), moderate (1000–9999 EPG), and heavy (≥10,000 EPG) for *T. trichiura*; light (1–99 EPG), moderate (100–399 EPG), and heavy (>400 EPG) for *S. mansoni* [15].

In STST, approximately three grams of fresh stool sample was weighed and homogenized in 10 ml of normal saline solution. The mixture was filtered through surgical gauze into a 50 ml falcon tube, and more saline solution was added up to 50 milliliters. Then, the falcon tube cap was tightly closed and shaked for 30 seconds and left to stand for 45 minutes. The supernatant was discarded; the sediment was taken by a Pasteur pipette, and duplicate smears were prepared and examined microscopically for any parasite stages detected from stool [19].

### 2.4. Quality Control

Before data collection, training was given to data collectors about data and stool sample collection protocols. Pretest was conducted among 22 participants (5% of the sample size) in order to check the validity of the questionnaire and laboratory-based data collection process. Standard operating procedures were followed during stool specimen collection, transportation, processing, examination, and recording. Each smear was examined by two laboratory technicians, and discordant results were resolved after discussion. Fortunately, no discordant results were reported between the two microscopists.

### 2.5. Data Analysis

Data were entered and analyzed using statistical package for the social sciences version 26. Prevalence of intestinal parasites and *S. mansoni* was analyzed with descriptive statistics. Intensity of STHs and *S. mansoni* infections was reported by the mean eggs counts per gram of stool. Factors associated with intestinal parasites and *S. mansoni* were first analyzed by univariate logistic regression. Variables with *p* value < 0.2 were adjusted by multivariate logistic regression to control the possible confounding factors. Variables were considered statistically significant if the *p* value < 0.05.

## 3. Results

### 3.1. Sociodemographic Characteristics

From the total sample size of 422, data from 388 (92%) participants was complete for analysis. The remaining 29 (6.8%) did not exist at their fishing site during data collection, and 5 (1.2%) did not provide stool samples. Age of study participants ranged from 14 to 50 years old with mean and standard deviation of 29.52 and 8.725, respectively. Majority of study participants, 306 (78.9%), live in rural settings while 194 (50.0%) attended primary school, and 272 (70.1%) had average monthly income of ≤1000 birr (Table 1).

### 3.2. Prevalence of Intestinal Parasites and *S. mansoni* Infections

Among 388 participants, 164 (42.3%) were tested positive for at least one species of parasites at least by one of the diagnostic methods used. Parasites were detected in samples from 68 (17.5%) and 141 (36.3%) participants by the KK and STST methods, respectively. Soil-transmitted helminths and intestinal protozoa were detected in 122 (31.4%) and 42 (10.8%) participants, respectively. *S. mansoni* was the most frequent parasite detected in 88 (22.7%) participants, followed by hookworms and *A. lumbricoides* with respective frequency of 76 (19.6%) and 46 (11.9%). Among 164 IP infected participants, 24 (14.6%) were coinfected by two parasite species; of these, hookworm and *A. lumbricoides* coinfections were predominantly detected in 9 (5.5%) participants. One Triple infection was detected in 1 (0.6%) participant with *E*. *histolytica/dispar*, hookworm, and *H. nana* (Table 2).

The highest prevalence of IPs was recorded among fishermen in Qorata 18 (58.0%) followed by Dek 26 (49.1%) kebeles. However, the lowest prevalence of intestinal parasites was obtained among fishermen in Debranta 13 (33.3%), Mirafe Mariam 6 (25.0%), and Lijomi 10 (32.7%) kebeles. The highest prevalence of *S. mansoni* infection was recorded among fishermen in Lijomi 10 (32.3%) followed by Dek 17 (32.0%) kebeles. However, the lowest prevalence of *S. mansoni* infection was obtained among fishermen in Mirafe Mariam 4 (16.7%) and Chicha 3 (12.5%) kebeles (Table 3).

### 3.3. Infection Intensity of STHs and *S. mansoni* among Fishermen

The prevalence of STHs and *S. mansoni* among fishermen using KK technique was 56 (14.4%) and 46 (11.9%), respectively. The mean *S. mansoni* egg count was 171.91 EPG. Among *S. mansoni* positives, 23 (50.0%) participants had light infection intensity while the remaining 16 (34.8%) and 7 (15.2%) had moderate and high infection intensity, respectively. The mean infection intensity of *A. lumbricoides*, hookworm species, and *T. trichiura* were 1352.15, 116.13, and 40 EPG, respectively. *Ascaris lumbricoides* had 25 (89.3%) light and 3 (10.7%) moderate infection intensities, but hookworm species and *T. trichiura* had only light infection intensity (Table 4).

### 3.4. Factors Associated with Intestinal Parasitic Infections among Fishermen

In the multivariate logistic regression, the odds of IPIs among fishermen who attended only primary school was 2.02-fold (AOR = 2.02; 95% CI: 1.16-3.55, *p* = 0.014) higher than those who have attended secondary school and above. The odds of IPI among fishermen who were illiterates was 2.54 times (AOR = 2.54; 95% CI: 1.35-4.77, *p* = 0.004) higher than those who have attended secondary school and above. The odds of IPI increased by 2.23 times (AOR = 2.23; 95% CI: 1.25-3.96, *p* = 0.007) in participants who do not wash hands before meal as compared to those who wash their hands before meal (Table 5).

### 3.5. Factors Associated with *S. mansoni* Infection among Fishermen

In the multivariate regression, the odds of *S. mansoni* infection among illiterate fishermen was 2.37-fold (AOR = 2.37; 95% CI: 1.13-4.95, *p* = 0.022) higher than those who have attended high school and above. Fishing by bargee increases the odds of *S. mansoni* infection by 2.08-fold (AOR = 2.43; 95% CI: 1.30-4.53, *p* = 0.005) than fishing by boat. The odds of *S. mansoni* infection among fishermen who were involved in fishing for ≥4 days per week was 2.27 times (AOR = 2.27; 95% CI: 1.09-4.75, *p* = 0.029) higher than those who fish for fewer days per week. The odds of *S. mansoni* infection among fishermen who had swimming habit in Lake Tana was 3.03-fold (AOR = 3.03; 95% CI: 1.11-8.25, *p* = 0.030) higher than those with no swimming habit. The odds of *S. mansoni* infection among fishermen who participated in irrigation was 3.09 times (AOR = 3.09; 95% CI: 1.80-5.31, *p* ≤ 0.001) higher than those who did not participate in irrigation (Table 6).

## 4. Discussion

Intestinal parasitic and *S. mansoni* infections are common problems in many developing countries including Ethiopia. In the present study, the overall prevalence of IPs among fishermen at Lake Tana was 42.3% which is higher than a previous result of 4.1% in Burkina Faso [21]. However, the present prevalence was lower than previous prevalence of 68.7% in Jimma town [22], 51.3% in Addiremets town [23], and 50.2% Abaye Deneba [24] all from Ethiopia. The difference in prevalence reports might be due to variations in age of study participants and sample size. About 54.7% and 48% of participants in studies from Jimma [22] and Abaye Deneba [24] were children, respectively, who are more exposed to IPs than adults due to poor sanitation.

Intestinal protozoan parasite infections are common in areas where poor sanitation and hygiene are practiced. In the present study, the prevalence of *E. histolytica/dispar and G. lamblia* was 7.5% and 3.6%, respectively. This is higher than a study finding in Addiremets town [23]. This might be due to differences in laboratory techniques as the previous study used direct microscopy whereas STST which has higher sensitivity is used in the present study.

The prevalence of STHs (31.4%) in the present study is higher than a study report of 26.5% in Addiremets town [23], but lower than prevalence of 67.3% from Bushulo village, southern Ethiopia [25]. The variation might be due to the difference in hygiene and sanitation that facilitates the transmission of the infection. The prevalence of hookworm (19.6%) in the present study was in consistent with prevalence of 23.1% in Addiremets town [23] but lower than findings of 43.3% from Uganda along the Lake Victoria [26]. On the other hand, hookworm prevalence in the present study was higher as compared to results from Hawassa (5.76%) [16] and Abaye Deneba (0.4%) [24]. These differences might be due to the variations in the frequency of soil contact and shoe wearing habit. Variations in the diagnostic approach might also be attributive for differences in prevalence across studies. For instance, three morning stool samples were collected and examined in the previous study from Uganda whereas single stool sample was collected and examined in the present study.

Infection frequency of *A. lumbricoides* (11.9%) in the present study was higher than study findings of 8.3% from Abaye Deneba [24] and 3.4% in Addiremets town [23], but lower than findings of 40.74% from Hawassa [16]. This difference might be due to variations in hygiene and sanitation of study participants and endemicity of *A. lumbricoides.*

Schistosomiasis is one of the most challenging parasitic infections in resource-limited countries. The prevalence of *S. mansoni* in the present study was 22.7% which is comparable with 26.3% prevalence from Addiremets town [23], 26.3% in Jimma [22], and 26.6% in Egypt [27]. This result was also higher than the results of 13.9% obtained from Brazil [28] and 16.35% in Burkina Faso [21], but lower than 29.21% prevalence in Hawassa [16], 41.3% in Abaye Deneba [24], 47.85% in Tanzania [29], 88.6% in Uganda Mayuge district [26], and 57.2% in Mukono district of Uganda [30]. The variation might be due to differences in laboratory techniques used, the variations in *S. mansoni* and snail distribution and fish catching places in Lakes. For instance in the present study, the majority of fishermen catch fish far from the border of the lake despite infection of cercaria mainly takes place near the border.

In the present study, the mean EPG of *A. lumbricoides* among fishermen was 1352.15 which was comparable with 1349.04 EPG from Hawassa [16], but lower than 5292 EPG in Addiremets town [23]. The mean EPG of hookworm species in the present study was also 116.13 which is higher than findings of 99.36 EPG from Hawassa [16] and lower than 563 EPG from Addiremets town [23]. The intensity of *S. mansoni* infection in the present study (171.91 EPG) is higher than previous findings of 158.88 EPG from Hawassa [16], but lower than 218 EPG from Addiremets town [23], 236.2 EPG from Uganda [26], and 183.21 EPG from Tanzania [29]. This difference might be due to differences in the endemicity parasites, treatment seeking behavior, and working environment.

In present study, being illiterate (*p* = 0.004) and attending only primary school (*p* = 0.014) were significantly associated with the prevalence of IPIs among fishermen. This report is in agreement with previous findings from Nahavand, Western Iran [31]. Infections of IPs among fishermen who did not wash their hands was also higher than those who washed their hands before meal, which is consistent with previous study reports [5, 32]. This can be justified as educated people will have better awareness about the transmission and prevention of IPs.

In the present study, *S. mansoni* infection among illiterate fishermen was higher than those who have attended high school and above. This finding is supported by a previous study report in Uganda which shows *S. mansoni* infection increased with declining education level [33]. This might be due to low level of knowledge or awareness on the transmission of *S. mansoni* among illiterates.

Schistosomiasis is a water-based disease transmitted by direct contact with cercaria infested water. Fishing by bargee increases the odds of *S. mansoni* infection by 2.08-fold than fishing by boat. This might be due to the possibility of frequent water contact while fishing. Because the bargee cannot protect water from squeezing to inside part of the bargee. In the same way, fishing ≥4 days per week and swimming habit in Lake Tana in this study were also significantly associated with *S. mansoni* infection which is similar to the previous findings reported from Hawassa, southern Ethiopia [16]. In the present study, the odds of *S. mansoni* infection among fishermen who participated in irrigation was 3.09 times higher than those who did not participate in irrigation. This finding is consistent with a study conducted in Hintallo-Wejerat, North Ethiopia [34]. This could be justified as our skin might be exposed to cercarial stage of *S. mansoni* during collecting water from the Lake and performing the irrigation activities with barefoot and bare hands.

Assessment of the IP and *S. mansoni* infection status among fishermen in Lake Tana for the first time is the strength of the present study, while failure in detecting the source of infection, sanitary status in and around the lake was the limitations.

In conclusion, the prevalence of IPs and *S. mansoni* infection is high among fishermen at Lake Tana. Most of the infections of *S. mansoni*, *A. lumbricoides*, *T. trichiura*, and hookworm species were with light intensity. Being illiterate or attending only primary education and having no hand washing habit before meal are significantly associated with IPIs. Being illiterate, fishing by bargee, fishing ≥4 days per week, swimming, and participating in irrigation activities are independent predictors of *S. mansoni* infection. Therefore, health education on IPs and *S. mansoni* infection transmission and prevention, and regular deworming to fishermen should be advocated among fishermen along the Lake Tana Basin. Further studies are recommended to assess the source of infection for fishermen and sanitary status around Lake Tana.

## Figures and Tables

**Table 1:** Sociodemographic characteristics of fishermen at Lake Tana, Northwest Ethiopia, 2021 (*N* = 388).

Variables		Number	Percent
Age group	<18	11	2.8
	18-40	316	81.4
	≥41	61	15.7

Residence	Urban	82	21.1
	Rural	306	78.9

Educational status	Illiterate	107	27.6
	Primary	194	50.0
	High school and above	87	22.4

Marital status	Single	166	42.8
	Married	222	57.2

Family size	<5	223	57.5
	≥5	165	42.5

Monthly income (in birr)	≤1000	272	70.1
	>1000	116	29.9

**Table 2:** The prevalence of intestinal parasites and *S. mansoni* infections among fishermen at Lake Tana, Northwest Ethiopia, 2021 (*N* = 388).

Parasite species		Prevalence [*n* (%)]			Prevalence at 95% CI
		KK	STST	Combined	
Helminths	Hookworm species	31 (7.9)	69 (17.8)	76 (19.6)	15.95-23.83
	A. *lumbricoides*	28 (7.2)	35 (9.0)	46 (11.9)	9.01-15.46
	*E*. *vermicularis*	1 (0.3)	1 (0.3)	2 (0.5)	0.14-1.87
	*S*. *stercoralis*	N/A	3 (0.8)	3 (0.8)	0.26-2.24
	T. *trichiura*	4 (1)	2 (0.5)	5 (1.3)	0.55-2.98
	Taenia spp.	3 (0.8)	4 (1.0)	5 (1.3)	0.55-2.98
	*H. nana*	4 (1)	6 (1.5)	9 (2.3)	1.23-4.35
	*S. mansoni*	46 (11.9)	54 (13.9)	88 (22.7)	18.61-27.18

Protozoans	*G. lamblia*	N/A	14 (3.6)	14 (3.6)	2.16-5.97
	*E. histolytica/dispar*	N/A	29 (7.5)	29 (7.5)	5.14-10.67

**Total positive participants**		**68 (17.5)**	**141 (36.3)**	**164 (42.3)**	**37.33-47.37**

^∗^ *N*: total examined; *n*: number of positive; N/A: not applicable.

**Table 3:** Prevalence of intestinal parasites and *S. mansoni* among fishermen across study sites bordering the Lake Tana, Northwest Ethiopia, 2021.

Data collection site		Total examined	IPs infection [*n* (%)]		*S. mansoni* [*n* (%)]	
			Positive	Negative	Positive	Negative
Bahir Dar Zuria district kebeles	Chicha	24	11 (45.8)	13 (54.2)	3 (12.5)	21 (87.5)
	Debranta	39	13 (33.3)	26 (66.7)	9 (23.0)	30 (77.0)
	Dek	53	26 (49.1)	27 (50.9)	17 (32.0)	36 (68.0)
	Lijomi	31	10 (32.7)	21 (67.7)	10 (32.3)	21 (67.7)
	Weramit	55	26 (47.3)	29 (52.7)	10 (18.2)	45 (81.8)
	Wonjeta	28	11 (39.3)	17 (60.7)	7 (25.0)	21 (75.0)
	Robit	31	14 (45.2)	17 (54.8)	6 (19.3)	25 (80.7)
	Sekelet	24	11 (45.8)	13 (54.2)	5 (20.8)	19 (79.2)

Dera district kebeles	Tana Dnbiso	48	18 (37.5)	30 (62.5)	10 (20.8)	38 (79.2)
	Mirafe Mariam	24	6 (25.0)	18 (75.0)	4 (16.7)	20 (83.3)
	Qorata	31	18 (58.0)	13 (42.0)	7 (22.6)	24 (77.4)

**Total**		**388**	**164 (42.3)**	**224 (57.7)**	**88 (22.7)**	**300 (77.3)**

**Table 4:** Infection intensity of STHs and *S. mansoni* among fishermen at Lake Tana, Northwest Ethiopia, 2021.

Parasite species	Infection intensity			
	Mean EPG	Light [*n* (%)]	Moderate [*n* (%)]	High [*n* (%)]
*S. mansoni*	171.91	23 (50.0)	16 (34.8)	7 (15.2)
*A. lumbricoides*	1352.15	25 (89.3)	3 (10.7)	0
Hookworm spp.	116.13	31 (100)	0	0
*T. trichiura*	40	4 (100)	0	0

**Table 5:** Univariate and multivariate analysis of factors associated with intestinal parasites among fishermen at Lake Tana, Northwest Ethiopia, 2021.

Associated factors		IPs infection		COR (95% CI)	*p* value	AOR (95% CI)	*p* value
		Pos.	Neg.				
Age group	<18	5	6	1.12 (0.31-4.08)	0.861		
	18-40	133	183	0.98 (0.56-1.70)	0.938		
	≥41	26	35	1			

Residence	Urban	32	50	1			
	Rural	32	174	1.19 (0.72-1.95)	0.503		

Educational status	Illiterate	57	50	3.17 (1.73-5.83)	≤0.001	2.54 (1.35-4.77)	0.004
	Primary	84	110	2.13 (1.22-3.70)	0.008	2.02 (1.16-3.55)	0.014
	Secondary school and above	23	64	1		1	

Marital status	Single	65	101	1			
	Married	99	123	1.25 (0.83-1.88)	0.283		

Family size	<5	92	131	1			
	≥5	72	93	1.10 (0.73-1.66)	0.639		

Monthly income	≤1000 birr	116	156	1.05 (0.68-1.64)	0.817		
	>1000 birr	48	68	1			

Latrine available	Yes	127	190	1		1	
	No	37	34	1.55 (0.92-2.59)	0.099	1.35 (0.78-2.34)	0.224

Latrine utilization	Yes	104	147	1			
	No	23	43	0.72 (0.41-1.26)	0.243		

Hand washing after toilet	Yes	91	130	1			
	No	13	17	1.09 (0.51-2.36)	0.822		

Water source for drinking	Tap	40	62	1			
	Untreated	124	162	1.19 (0.75-1.89)	0.468		

Hand washing before meal	Yes	125	200	1		1	
	No	39	24	2.60 (1.49-4.53)	0.001	2.23 (1.25-3.96)	0.007

Wash vegetables before eating	Yes	71	96	1			
	No	93	128	0.98 (0.65-1.48)	0.932		

**Table 6:** Univariate and multivariate analysis of factors associated with *S. mansoni* infection among fishermen at Lake Tana, Northwest Ethiopia, 2021.

Variables		*S. mansoni*		COR (95% CI)	*p* value	AOR (95% CI)	*p* value
		Pos.	Neg.				
Residence	Urban	13	69	1		1	
	Rural	75	231	1.72 (0.90-3.90)	0.099	0.77 (0.37-1.61)	0.492
Educational status	Illiterate	34	73	2.24 (1.12-4.45)	0.022	2.37 (1.13-4.95)	0.022
	Primary	39	155	1.21 (0.63-2.33)	0.574	1.42 (0.70-2.86)	0.329
	High school and above	15	72	1		1	
Fishing frequency per week	<4 days	11	74	1		1	
	≥4 days	77	226	2.29 (1.16-4.54)	0.017	2.27 (1.09-4.75)	0.029
Fishing instrument	Boat	19	114			1	
	Bargee	69	186	2.23 (1.27-3.89)	0.005	2.43 (1.30-4.53)	0.005
Swimming habit	No	5	47	1		1	
	Yes	83	253	3.08 (1.19-8.01)	0.021	3.03 (1.11-8.25)	0.030
Swimming frequency	Sometimes	53	70	1			
	Always	30	83	1.14 (0.68-1.93)	0.619		
Wash clothes in Lake Tana	No	2	9	1		1	
	Yes	86	281	2.92 (0.66-12.73)	0.157	1.87 (0.38-9.14)	0.439
Bathing in Lake Tana	No	2	18	1		1	
	Yes	2	282	2.75 (0.62-12.06)	0.181	1.97 (0.28-13.89)	0.495
Work in irrigation	No	26	184	1		1	
	Yes	62	116	3.78 (2.26-6.32)	≤0.001	3.09 (1.80-5.31)	≤0.001
Distance from border	≥1 km	53	161	1			
	<1 km	10	36	0.84 (0.39-1.82)	0.664		

## Data Availability

The original data for this study is available from the corresponding author.

## Abbreviations

AOR: Adjusted odds ratio
COR: Crude odds ratio
EPG: Egg per gram of stool
IPIs: Intestinal parasitic infections
IPs: Intestinal parasites
KK: Kato Katz
STH: Soil-transmitted helminths
STST: Spontaneous tube sedimentation technique.
